# Supplementary material for: Morbidity burden and predictors of hospitalization among unaccompanied migrants and persons prone to statelessness in Ghana
Source: PLOS Glob Public Health. 2026 Apr 17;6(4):e0006316. doi: 10.1371/journal.pgph.0006316 (PMC13089720; doi:10.1371/journal.pgph.0006316)
Supplement: S1 Text — (DOCX) [file pgph.0006316.s001.docx]

**Supplementary File: Data Collection Instrument (Questionnaire)**

**KWAME NKRUMAH UNIVERSITY OF SCIENCE OF TECHNOLOGY**

**COLLEGE OF ART AND BUILT ENVIRONMENT**

**FACULTY OF BUILT ENVIRONMENT**

**DEPARTMENT OF PLANNING**

**QUESTIONNAIRE FOR UNACCOMPANIED MIGRANTS AND PERSONS AT RISK OF STATELESSNESS**

**Brief background of the study**

This study seeks to examine the prevalent illnesses, frequency of hospital admissions, and the factors influencing their use of inpatient care among unaccompanied migrants and individuals prone to statelessness in Ghana.

This study is purely academic-oriented, and participation is voluntary. Participants may choose to withdraw from the study at any time or choose not to answer questions deemed private without any consequences. Your responses are confidential and will be used only for the reasons indicated above.

We kindly request your valuable time to participate in this study by answering a few questions. Your candid responses will be highly appreciated and improve the quality of the study.

Thank you very for agreeing to participate in this study.

**ELIGIBILITY CRITERIA**

| **SN** | **Question** | **Response** | **Code** |
| --- | --- | --- | --- |
|  | Are you below the age of eighteen (18) years? | No [**Skip to iii**]  Yes | 1[ ]  2[ ] |
|  | Have you lived outside your place of birth for more than six (6) months under the maintenance/care of other relatives other than a parent or any other person legally liable for your maintenance/care? | No  Yes | 1[ ]  2[ ] |
|  | Have you lived in Ghana for over five (5) years? | No [**End if i or ii is No**]  Yes | 1[ ]  2[ ] |
|  | Do you have a Ghanaian birth certificate, Ghanaian passport, National Identification Authority Card, or proof of citizenship in any state/country | No  Yes [**End if i or ii is No**] | 1[ ]  2[ ] |
|  | District of Residence | Greater Kumasi Metropolitan Area (GKMA)  Awutu Senya East Metropolitan Area (ASEMA) | 1[ ]  2[ ] |
|  | Community of Residence | Asawase  Asokwa  Bantama  Kwadaso  Manhyia  Nhyiaeso  Oforikrom  Suame  Subin  Tafo  Kasoa  Opeikuma  Adam Nana  Kpormertey  Ofaakor  Akweley  Walantu  Tuba  Kakraba  Amanfro | 1[ ]  2[ ]  3[ ]  4[ ]  5[ ]  6[ ]  7[ ]  8[ ]  9[ ]  10[ ]  11[ ]  12[ ]  13[ ]  14[ ]  15[ ]  16[ ]  17[ ]  18[ ]  19[ ]  20[ ] |
|  | Locality of Residence | Rural  Urban  Peri-urban/urban fringe  Slum | 1[ ]  2[ ]  3[ ]  4[ ] |

**SECTION A: SOCIODEMOGRAPHIC CHARACTERISTICS OF RESPONDENTS**

In this section, I seek to gain information about the demographic, social, and economic characteristics of respondents.

| **SN** | **Question** | **Response** | **Code** |
| --- | --- | --- | --- |
|  | Gender of Respondent | Male  Female | 1[ ]  2[ ] |
|  | Age of Respondent | Less than 18 years  19-24 years  25-29 years  30-34 years  35-39 years  40 or more | 1[ ]  2[ ]  3[ ]  4[ ]  5[ ]  6[ ] |
|  | Religion of Respondent | Christianity  Islam  African Traditional Religion  Others | 1[ ]  2[ ]  3[ ]  4[ ] |
|  | Marital Status of Respondent | Never married/single  Currently Married  Divorced/ separated.  Widowed | 1[ ]  2[ ]  3[ ]  4[ ] |
|  | Formal Education of Respondent | No formal education  Primary  Junior High School  Senior high school  Tertiary | 1[ ]  2[ ]  3[ ]  4[ ]  5[ ] |
|  | Language proficiency of Respondent | Akan/Twi  Dagaare/Waale  Ewe  Ga/Dangme  Gonja/Dagbani  Kasem  English  Other (specify)……… | 1[ ]  2[ ]  3[ ]  4[ ]  5[ ]  6[ ]  7[ ]  8[ ] |
|  | Are you living alone | No  Yes [**Skip to Q9**] | 1[ ]  2[ ] |
|  | What is the relationship with the person you live with? | Spouse  Parent  Son/Daughter  Other relatives (specify)  Friend/Neighbors  Church member  Other (specify) | 1[ ]  2[ ]  3[ ]  4[ ]  5[ ]  6[ ]  7[ ] |
|  | Employment Status of Respondent | Employed  Unemployed  Student  Retired  Others Specify……. | 1[ ]  2[ ]  3[ ]  4[ ]  5[ ] |
|  | How much income in Ghana cedis do you receive per month? *(1 dollar = 12.67 cedis at the time of data collection)* | 0-485  486-971  972-1457  1458-1943  1944 and above | 1[ ]  2[ ]  3[ ]  4[ ]  5[ ] |
|  | Are you enrolled on the national health insurance scheme (NHIS)? | No  Yes | 1[ ]  2[ ] |

**SECTION B: MORBIDITY AND HOSPITALIZATION**

In this section, I seek to gain information about incidence of illnesses, and frequency of hospital admissions among respondents.

| **SN** | **Question** | **Response** | **Code** |
| --- | --- | --- | --- |
|  | Have you been frequently ill? | Never  Less frequent  Frequent  Very frequent  Every day | 1[ ]  2[ ]  3[ ]  4[ ]  5[ ] |
|  | In general, how would you rate your overall health in the past 6 months? | Excellent  Very good  Good  Fair  Poor  Very poor | 1[ ]  2[ ]  3[ ]  4[ ]  5[ ]  6[ ] |
|  | Have you spent more than a complete week in bed at a health facility | Yes  No | 1[ ]  2[ ] |
|  | Have you been diagnosed with any chronic non- communicable illness? | No [**Skip to Q17**]  Yes | 1[ ]  2[ ] |
|  | Which of the chronic non-communicable illnesses were you diagnosed of? | Diabetes  Hypertension  Asthma  Cancers  Stroke  Chronic kidney disease  Depression  Arthritis  Insomnia  Mental disorder  Disorder of joint and bones  Eye problem  Ear problem | 1[ ]  2[ ]  3[ ]  4[ ]  5[ ]  6[ ]  7[ ]  8[ ]  9[ ]  10[ ]  11[ ]  12[ ]  13[ ] |
|  | Have you been diagnosed with any communicable illness? | No [**End**]  Yes | 1[ ]  2[ ] |
|  | Which of the communicable illness were you diagnosed with? (Tick all that apply) | Malaria  Cholera  Influenza (flu)/Common cold  Tuberculosis (TB)  Typhoid fever  Yellow fever  Hepatitis (A, B, C)  Measles/Chickenpox  STI/STD’s  Others, specify ……… | 1[ ]  2[ ]  3[ ]  4[ ]  5[ ]  6[ ]  7[ ]  8[ ]  9[ ]  10[ ] |
