# Supplementary material for: Morbidity burden and predictors of hospitalization among unaccompanied migrants and persons prone to statelessness in Ghana
Source: PLOS Glob Public Health. 2026 Apr 17;6(4):e0006316. doi: 10.1371/journal.pgph.0006316 (PMC13089720; doi:10.1371/journal.pgph.0006316)
Supplement: S1 Table — (DOCX) [file pgph.0006316.s002.docx]

**Table 5. Univariate complementary log–log regression of factors associated with hospitalization (N = 481)**

| **Variable Themes** | **Variables** | **OR** | **95% CI** | **p-value** |
| --- | --- | --- | --- | --- |
| Predisposing | Respondent Status | 0.840 | 0.511 - 1.382 | 0.492 |
|  | Sex | 0.713 | 0.371 - 1.372 | 0.311 |
|  | Marital Status | 1.388 | 0.842 - 2.288 | 0.198 |
|  | Religion | 0.798 | 0.489 - 1.301 | 0.365 |
|  | Formal Education | 0.815 | 0.437 - 1.522 | 0.521 |
|  | Age | 0.849 | 0.459 - 1.573 | 0.604 |
| Enabling | Insurance Coverage | 1.868 | 1.020 - 3.421 | 0.043 |
|  | Employment Status | 0.794 | 0.544 - 1.159 | 0.232 |
|  | District | 1.900 | 1.000 - 3.609 | 0.050 |
|  | Locality | 0.770 | 0.418 - 1.418 | 0.401 |
|  | Income | 1.240 | 0.442 - 3.477 | 0.682 |
|  | Social Network | 0.726 | 0.478 - 1.104 | 0.134 |
| Need | Illness Frequency | 5.963 | 3.130 - 11.358 | 0.000 |
|  | NCD | 5.412 | 2.763 - 10.601 | 0.000 |
|  | Infectious Disease | 2.605 | 1.413 - 4.802 | 0.002 |

**Notes**

1. OR = Odd Ratio derived from complementary log–log regression.
2. CI = Confidence Interval.
3. Hospitalization defined as ≥1-week inpatient stay.
4. Variables with p ≤ 0.05 were considered for multivariable analysis.
