# Supplementary material for: Morbidity burden and predictors of hospitalization among unaccompanied migrants and persons prone to statelessness in Ghana
Source: PLOS Glob Public Health. 2026 Apr 17;6(4):e0006316. doi: 10.1371/journal.pgph.0006316 (PMC13089720; doi:10.1371/journal.pgph.0006316)
Supplement: Attachment — Submitted filename: Response to Editors Comment_October_2025.doc [file pgph.0006316.s004.doc]

Handling Editor

PLOS Global Public Health

15 October 2025

**Dear Editor,**

I attach here the revised Manuscript with Ref: Submission ID PGPH-D-25-03075,

entitled, “**Morbidity Burden and Predictors of Hospitalization Among Unaccompanied Migrants and Persons Prone to Statelessness in Ghana**” On behalf of my coauthor, I write to thank you for your comments on the manuscript, and the opportunity granted us to resubmit a revised version for potential publication.

We hope that the revised manuscript will fulfil your expectations and will now be suitable for publication in your journal, **PLOS Global Public Health**

Thank you.

Yours sincerely,

*SIGNED*

**CORRESPONDING AUTHOR**

**AUTHORS’ RESPONSE TO EDITOR COMMENT(S)**

**Reviewer 1**

**Comment:** Please complete all items on the Human Participants Research Checklist that are relevant for your submission, by following this link: http://journals.plos.org/plosone/s/file?id=dc11/PLOSOne_Human_Subjects_Research_Checklist.docx. There may be overlap between the checklist items and other queries listed here; please address any duplicated queries both in your response email and on the checklist itself. Upload the completed Human Participants Research checklist as file type “Other” when you re-submit your manuscript. This document is for internal journal use only and will not be published if your article is accepted. The requested information will help us to assess whether your submission complies with PLOS’s policies and adheres to applicable reporting standards. Note that your manuscript may be rejected if you provide incomplete or inadequate responses to the checklist questions and that changing the ‘Section/Category’ of your article does not affect this requirement.

**Response:** Thank you so much for this comment. We have completed all items on the checklist that are relevant to our submission and uploaded the completed checklist as file type “Other”. To confirm that ethical approval was obtained for this study, we have uploaded the approval document from the ethics committee as file type “Other”. To clarify the start and end date of the recruitment period, we have revised the “Data and Method” section in the manuscript stating that “This study employed data from a cross-sectional survey conducted from March 26th to May 30th, 2024” (refer to line 116 – 117 of the revised manuscript). To clarify information on informed consent, we have revised the “Ethical Consideration” section in the manuscript stating that “Depending on contextual appropriateness and literacy level, written or verbal consent was obtained directly from non-minors but for minors under the care of relatives or guardians, consent was obtained from their designated caregivers. Verbal consent was specifically secured from participants unable to provide written consent. All verbal consent were witnessed and documented in the enumerator’s consent log” (refer to line 158 – 162 of the revised manuscript). We have also uploaded a sample of the written consent form as file type “Supporting Information”.
