# Supplementary material for: Morbidity burden and predictors of hospitalization among unaccompanied migrants and persons prone to statelessness in Ghana
Source: PLOS Glob Public Health. 2026 Apr 17;6(4):e0006316. doi: 10.1371/journal.pgph.0006316 (PMC13089720; doi:10.1371/journal.pgph.0006316)
Supplement: Attachment — Submitted filename: Plos_Response to Reviewers.doc [file pgph.0006316.s005.doc]

Handling Editor

PLOS Global Public Health

10 February 2026

**Dear Editor,**

I attach here the revised Manuscript with Ref: Submission ID PGPH-D-25-03075,

entitled, “**Morbidity Burden and Predictors of Hospitalization Among Unaccompanied Migrants and Persons Prone to Statelessness in Ghana**” On behalf of my coauthor, I write to thank you for your comments on the manuscript, and the opportunity granted us to resubmit a revised version for potential publication.

We hope that the revised manuscript will fulfil your expectations and will now be suitable for publication in your journal, **PLOS Global Public Health**

Thank you.

Yours sincerely,

*SIGNED*

**CORRESPONDING AUTHOR**

**AUTHORS’ RESPONSE TO EDITOR COMMENT(S)**

**Comment 1:** Please include a complete copy of PLOS’ questionnaire on inclusivity in global research in your revised manuscript. Our policy for research in this area aims to improve transparency in the reporting of research performed outside of researchers’ own country or community. The policy applies to researchers who have travelled to a different country to conduct research, research with Indigenous populations or their lands, and research on cultural artefacts. The questionnaire can also be requested at the journal’s discretion for any other submissions, even if these conditions are not met. Please find more information on the policy and a link to download a blank copy of the questionnaire here: https://journals.plos.org/globalpublichealth/s/best-practices-in-research-reporting. Please upload a completed version of your questionnaire as Supporting Information when you resubmit your manuscript.

**Response:** Thank you very much for this comment. We have completed the PLOS inclusivity in global research questionnaire and included a copy as Supporting Information in the revised submission.

**Comment 2:** Please ensure that your Ethics Statement is available in its entirety at the beginning of your Methods section, under a subheading 'Ethics Statement'.

**Response:** Thank you for this comment. We have revised the manuscript and placed the Ethics Statement at the beginning of the Methods section under the subheading “Ethics Statement.”

**Comment 3:** In the online submission form, you indicated that “Datasets for this study are available from the corresponding author upon reasonable request.”.

All PLOS journals now require all data underlying the findings described in their manuscript to be freely available to other researchers, either

1. In a public repository,

2. Within the manuscript itself, or

3. Uploaded as supplementary information.

This policy applies to all data except where public deposition would breach compliance with the protocol approved by your research ethics board. If your data cannot be made publicly available for ethical or legal reasons (e.g., public availability would compromise patient privacy), please explain your reasons by return email and your exemption request will be escalated to the editor for approval. Your exemption request will be handled independently and will not hold up the peer review process, but will need to be resolved should your manuscript be accepted for publication. One of the Editorial team will then be in touch if there are any issues.

**Response:** Thank you very much for this comment. The datasets used and/or analysed during the present study cannot be shared publicly because of **1**) ethical restrictions with the protocol approved by the ethics board for the study and **2**) the fact that it is coming from a broader PhD study which as per the requirements future publications are expected from it to meet the PhD requirements. The data can, however, be made available upon reasonable written request as indicated earlier. The contact details of the ethics committee that approved the conduct of this study are as follows: Committee on Human Research, Publication, and Ethics (CHRPE), School of Medicine and Dentistry, KNUST, University Post Office, Kumasi, Ghana. Email: [chrpe.knust.kath@gmail.com](mailto:chrpe.knust.kath@gmail.com) [/chrpe@knust.edu.gh](mailto:/chrpe@knust.edu.gh).

**Comment 3:** Some material included in your submission may be copyrighted. According to PLOS’s copyright policy, authors who use figures or other material (e.g., graphics, clipart, maps) from another author or copyright holder must demonstrate or obtain permission to publish this material under the Creative Commons Attribution 4.0 International (CC BY 4.0) License used by PLOS journals. Please closely review the details of PLOS’s copyright requirements here: PLOS Licenses and Copyright. If you need to request permissions from a copyright holder, you may use PLOS's Copyright Content Permission form.

Please respond directly to this email or email the journal office and provide any known details concerning your material's license terms and permissions required for reuse, even if you have not yet obtained copyright permissions or are unsure of your material's copyright compatibility.

Potential Copyright Issues:

a. Appendix 2 contains a logo. We are not permitted to publish this under our CC-BY 4.0 license, even with permission. We ask that you please remove or replace it.

If the reviewer comments include a recommendation to cite specific previously published works, please review and evaluate these publications to determine whether they are relevant and should be cited. There is no requirement to cite these works unless the editor has indicated otherwise.

**Response:** Thank you so much for this comment. We have removed the logo contained in Appendix 2 in accordance with the journals CC-BY 4.0 licensing requirements.

**AUTHORS’ RESPONSE TO REVIEWER COMMENT(S)**

**Reviewer 1**

**Comment 1:** This manuscript addresses an important and understudied topic: the morbidity burden and predictors of hospitalization among unaccompanied migrants and individuals prone to statelessness in Ghana. despite its relevance, the manuscript requires substantial revisions before it can be considered for publication.

**Response:** Thank you for the positive feedback. We are encouraged that the manuscript addresses an important and understudied topic in Ghana. We are grateful for opportunity to resubmit a revised version for potential publication.

**Comment 2:** Revise and justify the definition of Statelessness using established international frameworks (e.g., UNHCR, Ghana Nationality Act). Clarify how the study distinguishes undocumented but legally entitled individuals from those genuinely at risk of statelessness.

**Response:** Thank you very much for this comment. We have revised the definition of statelessness to align with UNHCR guidance (refer to lines 98 - 100 of the revised manuscript with track changes). We have also clarified the operational definition of statelessness to clearly delineate undocumented but legally entitled individuals from those genuinely at risk of statelessness (refer to lines 187 - 196 of the revised manuscript with track changes).

**Comment 3:** Hospitalization is defined as inpatient stay for more than one full week, which is inconsistent with global health services research (typically ≥24 hours). This definition excludes a large proportion of real hospitalizations, artificially depresses prevalence (8.7%), and may bias regression results. Revise the definition of Hospitalization or provide a strong methodological justification for using a 7-day threshold.

**Response:** Thank you very much for this comment. We acknowledge that global definitions often consider ≥24 hours; however, our use of a >7-day threshold was deliberate to capture clinically significant inpatient admissions and aid recall accuracy. This clarification has been incorporated in the method section of the revised manuscript (refer to lines 266 – 268 of the revised manuscript with track changes).

**Comment 4:** Present a clear sampling flowchart. Justify the selection of districts and communities. Explain how randomness was ensured at the participant level.

**Response:** Thank you for this comment. We have revised the manuscript to provide a clear flowchart of the sampling and justification for the selected districts. We have also clarified how participants were selection for inclusion in the study (refer to lines 181 – 185 of the revised manuscript with track changes).

**Comment 5:** Provide justification for each recoding decision as Variable Recoding reduces Analytical Precision, including statistical (model stability) or theoretical reasons.

**Response:** Thank you very much for the comment. We acknowledge that recoding reduces analytical precision, however due to rareness of the outcome variable, recoding was necessary to improve model stability. We have revised the manuscript to include justification for each recoding decision (refer to lines 272 – 294 of the revised manuscript with track changes).

**Comment 6:** Tables 2 and 3 show no statistically significant differences for any NCD or communicable disease across demographic and geographic subgroups (all p > 0.1). Despite this, the narrative repeatedly describes patterns as “higher,” “more common,” or “notable.” This is inappropriate and misleading. Rewrite the Results section to strictly reflect statistically supported findings. Where no significant differences exist, state this clearly and avoid narrative speculation based on raw counts.

**Response:** Thank you very much for this observation. We have revised the results section of the manuscript to strictly reflect statistically supported findings. Additionally, tables 2 and 3 have been revised to include the 95% confidence intervals (refer to lines 356 – 399 of the revised manuscript with track changes).

**Comment 7:** Regression tables lack 95% confidence intervals, which are mandatory for odds ratio interpretation. Include confidence intervals for all ORs in all models.

**Response:** Thank you very much for this comment. We have revised the regression table (table 4) to include the 95% confidence intervals for all Ors in all models (refer to lines 412 – 422 of the revised manuscript with track changes).

**Comment 8:** Interpretations across Models 1–3 are sometimes incorrect. For example, “prone to statelessness” is statistically significant in Model 2 but not in Model 3; however, the discussion presents it as a consistent predictor. Revise narrative to accurately reflect model-specific findings and note attenuation or loss of significance.

**Response:** Thank you very much for this observation. We have revised the narrative of table 4 to accurately reflect model-specific findings and highlighted the attenuation of significance across models (refer to lines 424 – 449 of the revised manuscript with track changes).

**Comment 9:** The Discussion section restates results extensively and occasionally presents speculative explanations (e.g., lifestyle shifts, informal care behaviors) that were not measured in the study. While Andersen’s Behavioral Model is cited, its application is superficial. The discussion does not meaningfully connect predisposing, enabling, and need factors to the results. Given that most subgroup differences were non-significant, this should be a central part of the Discussion, yet it is not addressed.

**Response:** Thank you very much for this comment. We have revised the discussion section to minimize the extensive statement of results, eliminate speculative explanations, and meaningfully connect predisposing, enabling and need factors. We have also addressed the inadvertent omission of the non-significant differences in subgroups. Refer to lines 476 – 544 of the revised manuscript with track changes).

**Comment 10:** Reframe policy recommendations to align directly with supported findings and emphasize caution due to limited generalizability.

**Response:** Thank you very much for this comment. We have revised the policy recommendations to align with supported findings and emphasize caution due to limited generalizability (refer to lines 548 – 593 of the revised manuscript with track changes).

**Comment 11:** While some limitations are acknowledged, several important ones are missing: Potential misclassification of statelessness, Highly restrictive hospitalization definition, Non-representative sampling from only two districts, Very small cell counts for diseases (1–11 cases in some categories), Instability of regression estimates due to sparse data. Expand the Limitations section to address these substantive concerns.

**Response:** Thank you very much for this observation. We have expanded the limitation section to address the concerns raised. Refer to lines 596 – 628 of the revised manuscript with track changes).

**Comment 12:** The Introduction is overly long, repetitive, and contains multiple overlapping paragraphs about migrant vulnerabilities. Reduce length by 30-40%, remove repetition, and sharpen the logical flow toward the research gap.

**Response:** Thank you for this comment. We have revised the introduction to enhance brevity, conciseness, and sharpen logical flow (refer to lines 53 – 123 of the revised manuscript with track changes).

**Comment 13:** Many interpretive comments appear in the Results section; others reappear in the exact same words in the Discussion. Ensure strict separation between empirical results and interpretation.

**Response:** Thank you very much for this observation. We have revised the results and discussion sections to strictly separate empirical results and interpretations.

**Reviewer 2**

**Comment 1:** In abstract, Methods section needs to be elaborated focusing on the flow of selection of these two areas along with participants, exposure and outcome assessment. Results section lacks frequency and percentages to get meaningful interpretation of the results.

**Response:** Thank you very much for this comment. We have revised the abstract to provide a clear flowchart of the sampling and selection of study areas. We have also revised the results section to aid meaningful interpretations (refer to lines 18 – 39 of the revised manuscript with track changes).

**Comment 2:** In Introduction, the authors have claimed disproportionate health risk faced by migrant population, but statistics related to it is missing in this section. (line number 42-45)

**Response:** Thank you very much for this comment. We have revised the Introduction to incorporate concise empirical evidence from prior studies demonstrating elevated health risks among migrant populations (refer to lines 58 – 59; 72 – 76 of the revised manuscript with track changes).

**Comment 3:** In line number 78-79, what SDG goal is affected from the objective authors claims to assess?

**Response:** Thank you for this comment. We clarified SDG goal affected from the objective assessed (refer to lines 112 – 115 of the revised manuscript with track changes).

**Comment 4:** In line number 83-85, the study objective doesn't inform inclusive healthcare policies and interventions. Rephrase the sentence and give the expected outcome of the study.

**Response:** Thank you very much for this comment. We have revised the manuscript to clarify the expected outcome of the study (refer to lines 119 – 121 of the revised manuscript with track changes).

**Comment 5:** The theoretical framework given from line number 86 to 110 can be included under methodology section.

**Response:** Thank you so much of this comment. We have revised the manuscript and inserted the theoretical framework under the Methods section.

**Comment 6:** In the line number 119-121, the authors have claimed the areas to be distinctive for target population, need some more information of the target population distribution in these areas and what does these areas represent as in organizational hierarchy?

**Response:** Thank you very much for this comment. We have revised the manuscript to clarify the justification for selection of the study areas (refer to lines 176 – 180 of the revised manuscript with track changes).

**Comment 7:** From line number 131-133, on what basis, the proportion of 0.5 was determined for sample size calculation? Sample size calculation for the determinants is missing.

**Response:** Thank you very much for this comment. We have revised the manuscript to include the basis for the determination and use of a 0.5 proportion in the sample size calculation (refer to lines 199 – 201 of the revised manuscript with track changes).

**Comment 8:** Operational definition of Persons Prone to Statelessness, Unaccompanied Migrants is not defined explicitly.

**Response:** Thank you very much for this comment. We have revised the manuscript to explicitly highlight the operationalization of persons prone to statelessness and unaccompanied migrants (refer to lines 187 – 196 of the revised manuscript with track changes).

**Comment 9:** In line number 202, the authors have mentioned three tier hierarchical regression analysis, what are the three tiers in that regression analysis?

**Response:** Thank you very much for this comment. We have revised the manuscript to clarify the regression approach adopted (refer to lines 307 – 312 of the revised manuscript with track changes).

**Comment 10:** The Inference statistics for primary objective is missing in the statistical analysis plan.

**Response:** Thank you very much for this comment. We have revised to manuscript to include the inference statistics for primary objective in the statistical plan (refer to lines 301 – 303 of the revised manuscript with track changes).

**Comment 11:** Information about the univariate regression and multivariable regression analysis is missing. How the variables were selected into the model is missing too.

**Response:** Thank you very much for this comment. We have revised the manuscript to clearly highlight how variables were selected into models (refer to lines 312 – 329 of the revised manuscript with track changes).

**Comment 12:** The interpretation given below the table 1 is repetition of information given in the table. Mention only the relevant findings of the table 1

**Response:** Thank you very much for this comment. We have revised interpretation of table 1 to focus only on relevant findings (refer to lines 335 – 344 of the revised manuscript with track changes).

**Comment 13:** The construction of Table 2 is poor, what does the p-value indicate? is the p-value specific to one self-reported morbidity status or it is for the entire morbidity status? Frequency for each morbidity is given, percentage is missing. 95% Confidence Interval for overall prevalence for each morbidity status is missing.

**Response:** Thank you very much for this comment. We have revised table 2 to clarity the p-values, include percentage and the 95% confidence interval for overall prevalence for each morbidity status (refer to lines 356 – 362 of the revised manuscript with track changes).

**Comment 14:** The comment 13 applies for table 3 too.

**Response:** Thank you very much for this comment. We have revised table 3 to clarity the p-values, include percentage and the 95% confidence interval for overall prevalence for each morbidity status (refer to lines 382 – 389 of the revised manuscript with track changes).

**Comment 15:** In table 4, what does Infrequent illness indicate, should be given in the table. The variables included in each model should be explicitly mentioned and the descriptive statistics about the hospitalization is missing in Table 4. Only the point estimate of Odds ratio is explicitly given, 95% CI is missing.

**Response:** Thank you very much for this comment. We have revised table 4 to improve clarity and include the 95% confidence interval for each odd ratio (refer to lines 412 – 422). We have also provided an explicit description of what constitutes infrequent illness under the measures section (refer to lines 292 – 294 of the revised manuscript with track changes).

**Comment 16:** Interpretation of the multivariable regression analysis for each covariates is wrong from line number 272 to 291.

**Response:** Thank you very much for this comment. We have revised the manuscript to clearly reflect the empirical findings (refer to lines 424 – 449 of the revised manuscript with track changes).
