# Supplementary material for: Morbidity burden and predictors of hospitalization among unaccompanied migrants and persons prone to statelessness in Ghana
Source: PLOS Glob Public Health. 2026 Apr 17;6(4):e0006316. doi: 10.1371/journal.pgph.0006316 (PMC13089720; doi:10.1371/journal.pgph.0006316)
Supplement: Attachment — Submitted filename: Response to Reviewers_01-03-2026.docx [file pgph.0006316.s006.docx]

Handling Editor

PLOS Global Public Health

1^st^ March 2026

**Dear Editor,**

I attach here the revised Manuscript with Ref: Submission ID PGPH-D-25-03075, entitled, “**Morbidity Burden and Predictors of Hospitalization Among Unaccompanied Migrants and Persons Prone to Statelessness in Ghana**” On behalf of my coauthor, I write to thank you for your comments on the manuscript, and the opportunity granted us to resubmit a revised version for potential publication.

We hope that the revised manuscript will fulfil your expectations and will now be suitable for publication in your journal, **PLOS Global Public Health**

Thank you.

Yours sincerely,

*SIGNED*

**CORRESPONDING AUTHOR**

**AUTHORS’ RESPONSE TO REVIEWER COMMENT(S)**

**Reviewer #1**

**Comment 1**: The definition of statelessness is grounded in the Ghana Citizenship Act, but it is not explicitly aligned with established international frameworks such as the 1954 Convention or UNHCR’s formal definition. The manuscript does not clearly situate its operational definition within global legal standards.

**Response**: Thank you very much for this comment. We acknowledge the need for clearer alignment with established international standards. We have revised the manuscript to explicitly situate the definition of statelessness within the 1954 Convention and UNHCR’s formal definition (refer to line 45; 80 - 81 of the revised manuscript with track changes). However, our operational definition (refer to lines 123 - 129) was deliberately formulated to ensure contextual precision and empirical measurability within the framework of Ghana’s nationality laws. This is consistent with UNHCR guidance, which emphasizes that determination of statelessness requires examination of the nationality laws of relevant States and their application in practice (1).

**Comment 2**: The hospitalization definition (>1 week inpatient stay) is justified on recall grounds, but no sensitivity analysis using the conventional ≥24-hour definition is provided. The manuscript does not empirically demonstrate how this restrictive threshold may have influenced prevalence or regression estimates.

**Response**: Thank you very much for this insightful observation. While ≥24-hour is the conventional threshold in defining hospitalisation, self-reported healthcare utilization varies in accuracy according to population characteristics, recall period, and event salience (2). In vulnerable populations, brief inpatient stays are particularly prone to recall decay and misclassification compared to longer admissions (3,4). We therefore used a >1-week threshold to prioritize specificity and capture severe inpatient morbidity in a setting without administrative validation. We have revised the measures section of the revised manuscript to provide empirical support for the restrictive threshold (refer to lines 201 – 204 of the revised manuscript with track changes). Additionally, we have acknowledged the restrictiveness of the threshold in the limitation section and indicated that it may have underestimated overall hospitalization prevalence and potentially affected regression estimates (refer to lines 451 – 456 of the revised manuscript with track changes).

**Comment 3**: A clear sampling flowchart is still missing. Although the text now clarifies purposive district selection and non-random inclusion, there is no visual presentation of participant screening, eligibility, and final inclusion.

**Response**: Thank you very much for this comment. A structured participant flow diagram has now been included to visually present district selection, eligibility assessment, exclusions, and final inclusion (refer to lines 152 – 166 of the revised manuscript with track changes). We have further revised the data and method section of the manuscript accordingly (refer to lines 136 – 137 of the revised manuscript with track changes).

**Comment 4**: The use of the term “multi-stage sampling” may remain misleading because the final participant inclusion was non-probability and inclusive rather than randomized. The terminology could still imply greater methodological rigor than was actually used.

**Response**: Thank you very much for this insightful comment. We have revised the methods section to indicate that a multi-stage sampling strategy was adapted. Furthermore, we have clarified in the methods that while sampling occurred in sequential stages, participant inclusion within the purposefully selected sites was non-probability and based on an inclusion criterion (refer to lines 115 – 119 of the revised manuscript with track changes). We have also explicitly acknowledged the implications for representativeness and generalizability in the limitations section to avoid overstating methodological rigor (refer to lines 456 – 457 of the revised manuscript with track changes)

**Comment 5**: Some residual narrative language in the Results section still uses comparative terms such as “more prevalent” or “higher” in contexts where statistical support is weak or exploratory. While improved, a few phrases could be further neutralized.

**Response**: Thank you very much for this insightful observation. We have carefully reviewed the results section and revised the remaining comparative language to ensure all statements are strictly aligned with statistical evidence, using neutral phrasing where associations were weak or exploratory.

**Comment 6**: The Discussion still contains speculative explanations (for example, sanitation or urban poverty mechanisms) that were not directly measured in the study. These interpretations are softer than before but remain somewhat inferential.

**Response**: Thank you for the comment. We have revised the Discussion to remove speculative language and instead situate the observed typhoid concentration and subgroup differences in NCD within established empirical evidence (refer to lines 379 - 394 of the revised manuscript with track changes)

**Comment 7**: The Introduction, although shortened, but Some repetition around migrant vulnerability remains.

**Response**: Thank you very much for this observation. We have revised the introduction to eliminate repetitive language while preserving conceptual coherence. The introduction now explicitly distinguishes structural exposure, healthcare access barriers, and broader social determinants of morbidity to foreground different dimensions of vulnerability among migrants without redundancy (refer to lines 46 – 72 of the revised manuscript with track changes).

**Comment 8**: Everything else, including regression interpretation corrections, confidence intervals, recoding justifications, expanded limitations, and improved model-specific interpretation, appears to have been adequately addressed.

**Response**: Thank you for the comment and for the careful assessment of the revisions. We appreciate your acknowledgment that the regression interpretation, confidence intervals, recoding justifications, expanded limitations, and model-specific interpretations have been adequately addressed.

**Reviewer #2**

**Comment 1**: Sample size calculation for the primary objective - proportion was taken from Sample Size Determination in Health Studies: A Practical Guide by Lwanga and Lemeshow. But, the guide recommends to use 0.5 as proportion if previous published literature is not available. So, the author claim that there is no published literature related to proportion of communicable and NCD among the selected study population? Sample size calculation for predictors of hospitalization is not explicitly stated.

**Response**: Thank you very much for this comment. The Lwanga and Lemeshow formula and the recommended proportion (0.5) was used solely to determine the adjusted minimum sample size (N = 444) for the study and did not influence the number of participants reporting specific conditions (refer to lines 132 – 134 of the revised manuscript with track changes). Prevalence estimates for NCDs (N = 39) and communicable diseases (N = 112) were calculated using respondents who reported a prior diagnosis as the numerator with the overall sample size as denominator. Condition-specific proportions on the other hand were derived from their respective responses (as numerator) with the respondents’ responses on prior diagnosis for NCD and communicable diseases (as denominator) based on the specific condition category. Furthermore, claim that no published literature related to proportion of communicable and NCD among the selected study population pertains to the Sub-Saharan African context (refer to lines 50 – 78 of the revised manuscript with track changes).

**Comment 2**: Operational definition of Persons Prone to Statelessness, Unaccompanied Migrants is not
defined explicitly.

**Response**: Thank you very much for this comment. We have revised the manuscript to explicitly situate the definition of statelessness and unaccompanied migrants within UNHCR’s formal definition (refer to line 43 – 45 and 79 - 81 of the revised manuscript with track changes). However, our operational definitions (refer to lines 120 - 131) were deliberately formulated to ensure contextual precision and empirical measurability within the framework of Ghana’s laws. This is consistent with UNHCR guidance, which emphasizes that determination of statelessness requires examination of the nationality laws of relevant States and their application in practice (1).

**Comment 3**: The Inference statistics for primary objective is still missing in the statistical analysis plan.

**Response**: Thank you very much for this insightful observation. We have revised the Analytic Framework section to explicitly incorporate inferential procedures for the primary objective (refer to lines 240 – 245 of the revised manuscript with track changes).

**Comment 4**: Line number 219-234 - The authors have given rationale for variable selection in description. But, it would be better to include Directed acyclic graphs (DAG) or statistical principle of variable selection in the multivariable regression model.

**Response**: Thank you very much for this insightful comment. While a formal DAG was not constructed, we have clarified that variable selection for the multivariable model was guided by univariate analyses (p ≤ 0.05) alongside theoretically and empirically justified covariates identified a priori. This approach, grounded in statistical and substantive considerations, has now been explicitly articulated in the revised methods section (refer to lines 253 – 276 of the revised manuscript with track changes).

**Comment 5**: Table 2 Prevalence and Sociodemographic Associations of NCD Among Respondents (N = 39) and Table 3 Prevalence and Sociodemographic Associations of Communicable Diseases Among Respondents (N = 112) along with their interpretation are not required as per your objective of interest. Univariate analysis should be performed with hospitalization as outcome of interest.

**Response**: Thank you very much for the insightful comment. However, we respectfully retain Tables 2 and 3 because they: 1) address the study’s first objective by characterizing the burden of communicable and non-communicable diseases (NCDs) and 2) characterises morbidity patterns among participants in alignment with the paper’s title. We acknowledge the value of your suggestions and have included a univariate analysis with hospitalization as the outcome to identify significant variables for the multivariate model as an appendix.

**Comment 6**: In Table 4, as per the frequency of outcome of interest i.e (hospitalization), it is advisable to include 4 to 5 variables in the model. But the authors have included more than the advisable predictor variables in the model.

**Response**: Thank you for this important methodological observation. While the traditional rule of thumb recommends a minimum of 10 events per variable (EPV), methodological evidence demonstrates that this criterion is not absolute. For instance, Vittinghoff and McCulloch (5) showed through simulation that acceptable bias and confidence interval coverage can be achieved with EPV below 10 under appropriate model conditions. Similarly, van Smeden et al (6) questioned the empirical foundation of rigid EPV rules, emphasizing that model performance depends more on overall design characteristics than on a fixed EPV cutoff. In our study, variable inclusion was guided by theoretical relevance and univariate evidence, with variables entered hierarchically in conceptually defined blocks. This is supported as a principled strategy for evaluating incremental explanatory contribution while limiting overfitting and preserving interpretability. We have clarified this justification in the revised manuscript and acknowledged the small event count as a limitation.
